# Systemic and Seasonal Drivers of Hospital Mortality: Revisiting the Early Learning Period Hypothesis

**DOI:** 10.7759/cureus.79125

**Published:** 2025-02-16

**Authors:** Nicolas Bakinde, Dokun Dairo, Deborah Ngo Bakinde, Marvin Crawford, Richard Snyder, Claudia Fotzeu

**Affiliations:** 1 Medicine, Morehouse School of Medicine, Atlanta, USA; 2 Medicine, Grady Memorial Hospital, Atlanta, USA; 3 Biological Sciences, Savannah State University, Savannah, USA; 4 Pulmonary and Critical Care Medicine, Grady Memorial Hospital, Atlanta, USA; 5 Medicine, Southern Regional Medical Center, Riverdale, USA

**Keywords:** covid-19 impact, early learning period (elp) hypothesis, healthcare system resilience, hospital mortality, mortality trends, quarterly mortality rates, resource allocation, seasonal factors, seasonal mortality patterns, systemic factors

## Abstract

Introduction and background

The Early Learning Period (ELP) hypothesis posits that hospital mortality increases during the early academic months, traditionally attributed to transitional challenges such as trainee inexperience and changes in care teams. Understanding the validity of this hypothesis is crucial for guiding healthcare strategies, either toward trainee-focused reforms if validated or systemic interventions if refuted. However, systemic and seasonal factors, such as winter respiratory illness surges and healthcare resource strain, may play a more significant role in hospital mortality trends.

Methods

This was a retrospective observational study utilizing the 2021 National Inpatient Sample (NIS), a nationally representative database covering approximately 20% of U.S. hospitalizations. The study analyzed 5.6 million adult hospitalizations from 2021, excluding pediatric cases and records with missing mortality data. Hospital mortality trends were compared quarterly (Q1: January-March, Q2: April-June, Q3: July-September, Q4: October-December) to evaluate associations with seasonal and systemic factors.

Results

Contrary to the ELP hypothesis, hospital mortality was highest in Q1 (4.0%), consistent with seasonal factors like winter illnesses, and lowest in Q2 (2.7%). Mortality in Q3 (3.6%), the period associated with new trainee arrivals, was lower than in Q1.

Conclusion

This study refutes the ELP hypothesis, demonstrating that systemic and seasonal factors, rather than trainee inexperience, primarily drive hospital mortality trends. Proactive resource allocation targeted at seasonal drivers, particularly during high-demand periods such as Q1, is crucial to improving patient outcomes. These findings emphasize the need for systemic interventions, including enhanced resource allocation and flexible staffing models, rather than trainee-centered reforms. Future research should incorporate monthly mortality trends and teaching hospital-specific data for a more comprehensive understanding.

## Introduction

The Early Learning Period (ELP) hypothesis posits that hospital mortality increases during the early academic year, commonly attributed to trainee inexperience and organizational inefficiencies in teaching hospitals. This phenomenon, colloquially known as the "July Effect," suggests that the influx of newly graduated trainees and the rotation of experienced staff during the start of the academic year leads to lapses in patient safety and adverse clinical outcomes. While "July" is often referenced as a symbolic marker of these transitions, the broader hypothesis extends to patterns observable during the initial quarter of the academic year.

Despite its widespread perception, empirical support for the ELP hypothesis has been inconsistent. Early studies suggested higher mortality and complication rates in teaching hospitals during the summer months [1,2]; however, more recent analyses have challenged these findings, attributing seasonal mortality variations to systemic factors such as influenza prevalence, resource constraints, and patient case mix [3-5]. While teaching hospitals often experience greater clinical complexity, non-teaching hospitals have demonstrated comparable seasonal variations in mortality, suggesting that factors beyond trainee turnover, such as systemic and environmental influences, may play a more substantial role in driving these trends [6].

This study examines nationwide hospital mortality trends by quarter to critically evaluate the validity of the ELP hypothesis. By leveraging a large, nationally representative dataset, this analysis distinguishes systemic factors, such as seasonal illnesses and regional resource allocation, from potential trainee-related influences. These findings aim to inform more targeted interventions that enhance hospital outcomes year-round while challenging the conventional narrative surrounding the "July Effect."

## Materials and methods

Study design and data source

This retrospective observational study utilized data from the National Inpatient Sample (NIS), a database developed as part of the Healthcare Cost and Utilization Project (HCUP), Agency for Healthcare Research and Quality (AHRQ) [7]. The NIS is the largest publicly available all-payer inpatient healthcare database in the United States, capturing data on approximately 20% of all U.S. hospitalizations annually. This study specifically analyzed the 2021 NIS dataset, which covers a full calendar year, ensuring a comprehensive evaluation of quarterly hospital mortality trends. The dataset was weighted to produce national estimates, facilitating robust comparisons across discharge quarters.

Inclusion criteria

The study population included adult patients aged 18 years and older with complete mortality outcome data. All hospital types represented within the NIS database were considered.

Exclusion criteria

The exclusion criteria comprised pediatric admissions (patients under 18 years of age), hospitalizations with missing or incomplete mortality data, and records with data inconsistencies or errors in discharge coding.

Study variables

The study analyzed quarterly in-hospital mortality trends using patient- and hospital-level characteristics derived from the 2021 NIS. Discharges were categorized by calendar quarters to align with seasonal patterns. The independent variables included patient demographics, clinical risk classifications, and hospital-level attributes. The primary outcome was in-hospital mortality, defined as death during the index hospitalization. Details of the variables are presented in Table 1 below.

**Table 1:** Study variables and discharge quarter categories *Index Hospitalization: Refers to the hospital admission under investigation during which patient outcomes were measured. This excludes readmissions or subsequent hospitalizations and focuses on the initial admission for outcome assessment. APRDRG: All Patient Refined Diagnosis Related Group

Category	Variables
Discharge Quarters	Q1: January–March; Q2: April–June; Q3: July–September; Q4: October–December
Patient-Level Characteristics (Independent Variables)	Age (continuous); Sex; Race/Ethnicity: White, Black, Hispanic, Other; APRDRG Mortality Risk Classification: minor, moderate, major, extreme
Hospital-Level Characteristics (Independent Variables)	Admission Type: Elective vs. Non-elective; Geographic Region: Nine U.S. Census Divisions; Discharge Quarter
Primary Outcome	In-hospital mortality (death during index hospitalization*)
Secondary Outcome	None (This study focused solely on primary outcome trends)

Due to the significant portion of missing data (over 90%) for teaching hospital status, this variable was excluded from the analysis. This limitation restricted the ability to directly compare teaching and non-teaching hospitals, but the study aimed to provide a broad assessment of systemic mortality trends.

Statistical analysis

Baseline characteristics of the study population were summarized using descriptive statistics, stratified by discharge quarter. Differences between quarters were assessed using Chi-square tests for categorical variables and ANOVA for continuous variables, with statistical significance set at p < 0.05.

Multivariable logistic regression models were constructed to evaluate the association between discharge quarter and in-hospital mortality, adjusting for patient- and hospital-level covariates. Odds ratios (ORs) with 95% confidence intervals (CIs) were reported for each variable. Interaction terms assessed the modifying effects of age and admission type. Model diagnostics, including the Akaike Information Criterion (AIC) and pseudo-R², were used to evaluate goodness-of-fit and robustness.

All statistical analyses were performed using SPSS version 27 (IBM Corp., Armonk, NY, USA). Statistical significance was defined as a two-tailed p-value < 0.05.

Ethical considerations

The study utilized de-identified data from a publicly available database and was exempt from institutional review board (IRB) approval per federal guidelines.

## Results

Demographics and clinical characteristics

A total of 5.6 million hospitalizations from 2021 were analyzed, spanning all U.S. hospital types. The baseline demographic and clinical characteristics varied slightly across quarters. Patients in Q1 (January-March) were younger on average (64.8 ± 15.3 years) compared to Q3 (66.0 ± 15.0 years). Sex distribution was balanced across all quarters, with females representing 51.5% of the population. Minimal variation was observed in racial/ethnic composition and regional distribution of hospitalizations (Table 2).

**Table 2:** Baseline Characteristics of Study Population Q1: January–March; Q2: April–June; Q3: July–September; Q4: October–December; APRDRG: All Patient Refined Diagnosis Related Groups

Variable Category	Overall (%)	Q1 (%)	Q2 (%)	Q3 (%)	Q4 (%)
Demographic Characteristics					
Age (Mean ± SD)	65.4 ± 15.2	64.8 ± 15.3	65.0 ± 15.1	66.0 ± 15.0	65.8 ± 15.3
Male (%)	48.5	48.0	48.2	49.0	48.8
Female (%)	51.5	52.0	51.8	51.0	51.2
Race/Ethnicity					
White (%)	65.0	64.5	64.8	65.5	65.2
Black (%)	20.1	20.2	20.0	20.3	20.1
Hispanic (%)	10.5	10.4	10.6	10.2	10.7
APRDRG Mortality Risk					
Minor Risk (%)	40.7	40.0	40.5	41.2	40.8
Moderate Risk (%)	22.5	23.0	22.8	22.2	22.4
Major Risk (%)	21.5	22.0	21.3	21.6	21.4
Extreme Risk (%)	15.2	15.0	15.4	15.0	15.4
Geographic Region					
New England (%)	4.8	4.7	4.8	4.9	4.8
Middle Atlantic (%)	13.4	13.2	13.3	13.5	13.4
East North Central (%)	15.1	15.0	15.2	15.3	15.1
West North Central (%)	6.7	6.6	6.7	6.8	6.7
South Atlantic (%)	21.5	21.2	21.3	21.7	21.4
East South Central (%)	6.8	6.7	6.9	7.0	6.8
West South Central (%)	12.1	12.0	12.2	12.3	12.1
Mountain (%)	6.3	6.1	6.4	6.5	6.3
Pacific (%)	13.3	13.0	13.4	13.5	13.3
Admission Characteristics					
Weekend Admission (%)	25.0	24.3	25.1	25.5	24.9
Weekday Admission (%)	75.0	75.7	74.9	74.5	75.1

Mortality outcome and descriptive findings

**Figure 1:**
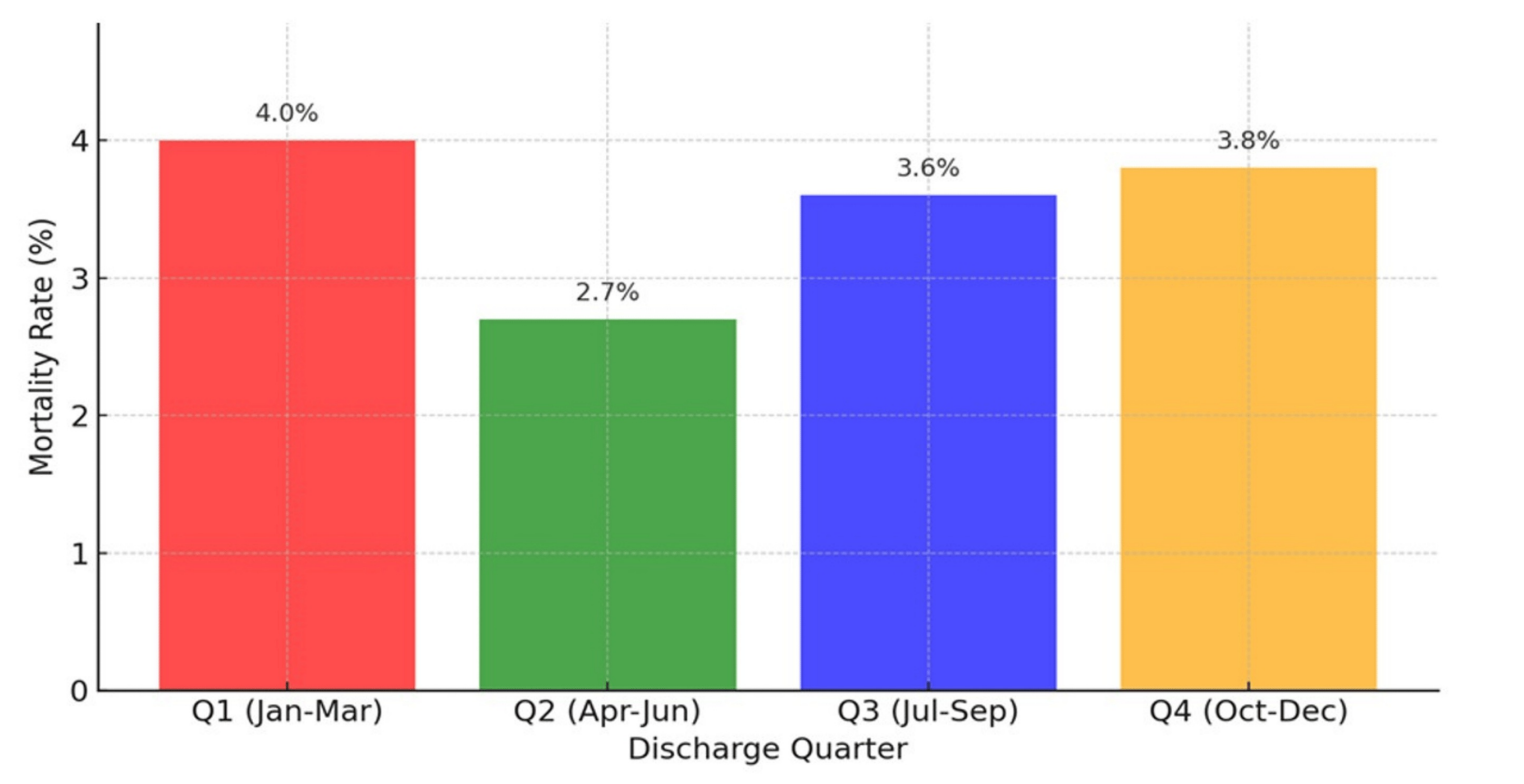
Mortality Trends by Quarter

Mortality rates were highest in Q1 (4.0%) and lowest in Q2 (2.7%), reflecting the impact of systemic strain and winter illnesses during the early part of the year (Table 3). Q3, which coincides with the start of the academic year and the arrival of new trainees, showed a mortality rate of 3.6%. Figure 1 visually illustrates these trends, showing the seasonal fluctuations in mortality rates across quarters.

**Table 3:** Mortality Rates by Quarter Q1: January–March; Q2: April–June; Q3: July–September; Q4: October–December

Mortality Outcome	Q1	Q2	Q3	Q4	Total
Survived	1,324,689 (96.0%)	1,395,820 (97.3%)	1,397,201 (96.4%)	1,360,641 (96.2%)	5,478,351 (96.5%)
Died	55,793 (4.0%)	38,149 (2.7%)	51,619 (3.6%)	53,959 (3.8%)	199,520 (3.5%)
Total Admissions	1,380,482	1,433,969	1,448,820	1,416,600	5,677,871

Quarterly mortality trends

Multivariable logistic regression identified significant predictors of in-hospital mortality, including discharge quarter, patient age, and All Patient Refined Diagnosis Related Groups (APRDRG) risk classification. Mortality was highest in Q1 (January-March) and lowest in Q3 (July-September). Using Q1 as the reference, the odds ratios for in-hospital mortality across quarters were: Q2: OR = 0.928 (95% CI: 0.912-0.945, p < 0.001); Q3: OR = 0.909 (95% CI: 0.894-0.926, p < 0.001); Q4: OR = 0.941 (95% CI: 0.924-0.958, p < 0.001).

Increasing age and extreme APRDRG risk were also strong predictors of mortality. Extreme APRDRG risk was associated with a seven-fold increase in odds (OR = 7.679, p < 0.001), and each additional year of age was associated with a modest increase in risk (OR = 1.073, p < 0.001) (Table 4).

**Table 4:** Logistic Regression Results Odds ratios (Exp(B)) indicate the relative likelihood of mortality for each variable, with statistical significance determined at p < 0.05. Q1: January–March; Q3: July–September; APRDRG: All Patient Refined Diagnosis Related Groups

Variable	B (Coefficient)	S.E. (Standard Error)	Wald Statistic	df	p-value (Sig.)	Exp(B) (Odds Ratio)
Discharge Quarter						
Q1 (Reference)	-	-	-	-	-	1.000
Q3 vs. Q1	-0.092	0.013	139.127	1	<0.001	0.909
Age (Years)	0.071	0.002	1,672.846	1	<0.001	1.073
Age × Quarter Interaction	Significant	-	-	-	<0.001	-
APRDRG Risk Classification						
Minor Risk (Reference)	-	-	-	-	-	1.000
Extreme Risk vs. Minor Risk	2.039	0.016	15,554.415	1	<0.001	7.679
Census Division						
East South Central (Reference)	-	-	-	-	-	1.000
South Atlantic	0.086	0.014	38.876	1	<0.001	1.090
Race						
White (Reference)	-	-	-	-	-	1.000
Black	0.174	0.016	118.733	1	<0.001	1.190
Elective Admission						
Non-Elective (Reference)	-	-	-	-	-	1.000
Elective	-0.054	0.012	20.166	1	<0.001	0.947
Weekend Admission	0.206	0.011	351.273	1	<0.001	1.229
Constant	-0.601	0.016	1,453.217	1	<0.001	0.548

These findings underscore the importance of proactively identifying high-risk patients, particularly those with extreme APRDRG risk, to mitigate mortality during peak demand periods.

**Figure 2:**
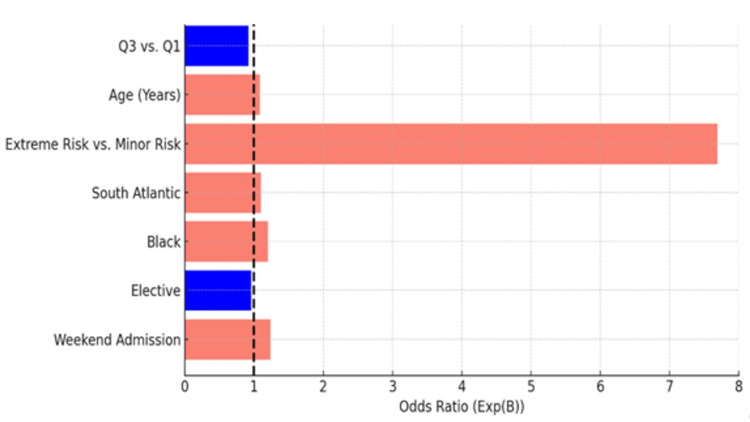
Logistic regression results Q1: January–March; Q3: July–September

Figure 2 provides a visual summary of these logistic regression results, highlighting the predictors of in-hospital mortality, including discharge quarter and APRDRG risk.

Age-related interactions

Older adults (≥65 years) experienced disproportionately higher mortality during Q1 compared to younger groups (interaction p < 0.001). These findings underscore the need for targeted interventions to address the unique risks faced by older adults during high-mortality periods, particularly in Q1.

Sensitivity analyses

Sensitivity analyses excluding hospitals with incomplete data on key variables confirmed the robustness of the findings. AIC and pseudo-R² values demonstrated good model fit and predictive accuracy, further validating the logistic regression model.

## Discussion

Interpretation of findings

The observed increase in mortality during Q1 aligns with the well-documented seasonal upticks in hospital admissions due to respiratory illnesses such as influenza [3-5]. These conditions strain healthcare systems, exacerbating existing resource shortages [8]. Further complicating this issue are regional disparities in healthcare infrastructure, which intensify the challenges during high-demand periods, as evidenced by variations in hospital capacity and outcomes across different geographic regions [9]. This increase in resource demand during winter months notably heightens the risk of adverse events, underscoring the urgent need for enhanced systemic preparedness [10]. Conversely, the absence of increased mortality in Q3 suggests that trainee transitions have minimal impact on patient outcomes. This undermines the assumption that inexperienced trainees during the academic year's start are a primary cause of increased mortality [5].

Seasonal versus trainee factors

The findings suggest that winter illnesses, resource strain, and systemic challenges are primary drivers of hospital mortality, particularly in Q1. This calls into question the assumption that the presence of inexperienced trainees during Q3 has a significant negative impact on mortality. The absence of a notable increase in mortality during Q3, a period traditionally associated with the influx of new trainees, undermines the commonly held belief that these trainees significantly compromise patient outcomes. Instead, it appears that systemic and seasonal factors play a more pivotal role in fluctuating mortality rates, highlighting the need for targeted systemic interventions during peak demand periods.

COVID-19 as a confounder

The COVID-19 pandemic heavily influenced healthcare delivery in 2021, adding significant complexity to mortality trends. The diversion of hospital resources toward managing COVID-19 cases likely impacted the availability and quality of care for other conditions, potentially inflating mortality rates across various non-COVID conditions [11].

Additionally, the pandemic's effect on staffing, particularly through burnout and sickness among healthcare workers, could have indirectly influenced mortality rates. High stress and reduced staffing levels are known to affect patient care quality, which in turn could contribute to higher mortality rates [12]. These factors may have exacerbated systemic challenges such as resource shortages, obscuring traditional seasonal variations [13].

Hospitals experienced variations in COVID-19 caseloads, which may have led to inconsistent impacts across different regions and types of hospitals, complicating the task of distinguishing pandemic-specific effects from other seasonal and systemic factors influencing hospital mortality [14].

Analyzing these pandemic-specific variables is crucial for a comprehensive understanding of the 2021 mortality data. Disentangling these effects from broader patterns requires a nuanced approach that considers both the direct impacts of the virus on patients and its systemic effects on healthcare delivery. Further analyses should aim to isolate these pandemic-related variables, possibly through comparative studies with previous years where such pandemic effects were not present, to accurately assess the true impact of systemic healthcare factors and the direct effects of COVID-19 on hospital mortality [15].

Evolving medical training landscape

The traditional boundaries defining teaching hospitals are becoming increasingly fluid. More hospitals that are not officially designated as teaching institutions are hosting residents, driven by expanding training needs, partnerships with medical schools, and broader healthcare system integrations. These shifts reflect a global trend towards more integrated and flexible healthcare training environments [16].

Additionally, the distinctions between university-based and community-based residency programs, and their impact on the quality of medical training, have been examined, adding depth to our understanding of these complexities [17]. As Cox and Desai point out, the growing need for diverse training settings is addressing a broad crisis in clinical education, highlighting challenges and opportunities within the current systems of medical training [18].

This evolving diversity in training settings is reshaping where and how medical training occurs, potentially affecting the data on hospital teaching status and complicating direct comparisons between traditionally defined teaching and non-teaching hospitals.

Limitations and impact of missing teaching hospital data

While this study benefits from the comprehensive, nationally representative 2021 NIS dataset, it is not without limitations that must be acknowledged. A notable limitation is the extensive missing data for teaching hospital status, which affects over 90% of our dataset. This substantial gap precludes a direct comparison between teaching and non-teaching hospitals, a factor potentially critical for dissecting the nuances of the ELP hypothesis. Consequently, our findings must be interpreted with caution, recognizing that they may not fully capture the variations in hospital practices and cultures that could influence mortality rates.

Furthermore, while sensitivity analyses or multiple imputation are commonly employed to assess and mitigate the impact of missing data [19], these methods presuppose that the missing data are not substantial enough to bias the imputed values significantly. In cases where missing data constitutes a small to moderate proportion of the dataset, these techniques can be very effective. However, the variable for teaching hospital status in this study had over 90% missing data, making methods such as multiple imputation impractical - a limitation further explained in the following discussion.

At such high levels of missingness, the assumptions underpinning multiple imputation, particularly the assumption of missing at random (MAR), may not hold. This limitation is highlighted by research indicating that high levels of missing data can significantly affect the performance of multiple imputation, leading to biased and unreliable outcomes [20]. In situations like ours, where the proportion of missing data is exceptionally high, the reliability of statistical estimates obtained through imputation significantly decreases, potentially introducing more bias into the results rather than mitigating it. Therefore, we opted not to use these methods but acknowledge that this decision limits our ability to explore how differences between teaching and non-teaching hospitals might affect mortality rates.

Additionally, a significant limitation of this study is its focus solely on in-hospital mortality, excluding post-discharge deaths. This approach may underestimate overall mortality trends, as patients discharged early or transferred to other facilities could experience mortality outcomes outside the hospital setting. Consequently, our findings may not fully represent patient outcomes beyond the hospital stay, particularly for high-risk populations. Future research incorporating longitudinal follow-up data or linkage with post-discharge outcomes could provide a more comprehensive assessment of mortality patterns.

Implications for practice and future directions

The data underscore the necessity for systemic interventions rather than focusing solely on trainee-related reforms. Proactive measures to manage peak demands during winter months, such as optimizing staffing flexibility, enhancing resource allocation, and boosting overall hospital preparedness, could markedly improve patient outcomes. Additionally, promoting continuous team integration and robust supervision throughout the year could alleviate systemic inefficiencies and elevate care quality [21].

Future studies should aim to secure datasets enriched with detailed teaching hospital data and account for monthly trends to more accurately discern patterns in hospital mortality. Incorporating controls for pandemic-related variables will also be crucial for a clearer understanding of the dynamics influencing hospital mortality. Expanding data collection efforts to capture these critical factors will allow researchers to formulate more precise and context-specific healthcare recommendations, highlighting the ongoing need for improvements in data collection methods and healthcare information systems.

## Conclusions

This study refutes the ELP hypothesis, demonstrating that systemic and seasonal factors, such as winter illnesses and resource strain, rather than trainee inexperience, primarily drive hospital mortality trends. The findings emphasize that mortality peaks in Q1 (January-March), coinciding with well-documented seasonal pressures, while Q3 (July-September), traditionally linked to new trainee arrivals, does not show increased mortality.

Although the study was limited by the absence of comprehensive teaching hospital data, which precluded direct comparisons between teaching and non-teaching hospitals, the results strongly suggest that systemic factors, not academic transitions, are the predominant contributors to mortality trends.

These findings underscore the need for healthcare systems to prioritize systemic interventions, including improved resource allocation and flexible staffing during peak demand periods. Future research should incorporate monthly mortality patterns, detailed teaching hospital data, and pandemic-specific adjustments to further refine our understanding of hospital mortality drivers.
